# Bile acids attenuate PKM2 pathway activation in proinflammatory microglia

**DOI:** 10.1038/s41598-022-05408-3

**Published:** 2022-01-27

**Authors:** Lorenzo Romero-Ramírez, Concepción García-Rama, Siyu Wu, Jörg Mey

**Affiliations:** 1 Laboratorio de Regeneración Nerviosa e Inmunidad Innata, Hospital Nacional de Parapléjicos, SESCAM, Finca la Peraleda s/n, 45071 Toledo, Spain; 2 School of Mental Health and Neuroscience and EURON Graduate School of Neuroscience, Maastricht University, Maastricht, The Netherlands

**Keywords:** Neuroscience, Glial biology, Neuroimmunology

## Abstract

Glycolysis is the metabolic pathway that converts glucose into pyruvate. Central nervous system (CNS) pathologies, such as spinal cord injury (SCI) and ischemia, are accompanied by an increase of the glycolytic pathway in the damaged areas as part of the inflammatory response. Pyruvate kinase is a key glycolytic enzyme that converts phosphoenolpyruvate and ADP to pyruvate and ATP. The protein has two isoforms, PKM1 and PKM2, originated from the same gene. As a homodimer, PKM2 loses the pyruvate kinase activity and acts as a transcription factor that regulates the expression of target genes involved in glycolysis and inflammation. After SCI, resident microglia and hematogenous macrophages are key inducers of the inflammatory response with deleterious effects. Activation of the bile acid receptor TGR5 inhibits the pro-inflammatory NFκB pathway in microglia and macrophages. In the present study we have investigated whether bile acids affect the expression of glycolytic enzymes and their regulation by PKM2. Bacterial lipopolysaccharide (LPS) induced the expression of PKM1, PKM2 and its target genes in primary cultures of microglial and Raw264.7 macrophage cells. SCI caused an increase of PKM2 immunoreactivity in macrophages after SCI. Pretreatment with tauroursodeoxycholic acid (TUDCA) or taurolithocholic acid (TLCA) reduced the expression of PKM2 and its target genes in cell cultures. Similarly, after SCI, TUDCA treatment reduced the expression of PKM2 in the lesion center. These results confirm the importance of PKM2 in the inflammatory response in CNS pathologies and indicate a new mechanism of bile acids as regulators of PKM2 pathway.

## Introduction

CNS pathologies, such as traumatic injuries and ischemia, evoke an immediate inflammatory response as a defense mechanism^1^. As a consequence of the injury, dead cells, cellular debris, cytokines and other proinflammatory mediators are released into the neural parenchyma, activating CNS resident macrophages (microglia)^1^. In addition to the morphological changes, activated microglial cells increase their migratory capacity toward the lesion site, raising their phagocytic activity and secreting inflammatory mediators^2^. Hematogenous macrophages are attracted by these inflammatory mediators invading the neural parenchyma and mediating additional phagocytosis and inflammatory response^3^. Proinflammatory macrophages are responsible for the chronification of the inflammation with detrimental effects on neuronal survival^4^. Induction of antiinflammatory macrophages phenotype would be more desirable to induce the resolution of the inflammatory response as well as tissue regeneration^5^.

Proinflammatory macrophages have a high demand for energy and biosynthetic precursors (e.g. proteins, lipids and nucleic acids). The metabolic changes that undergo proinflammatory macrophages resemble the Warburg effect described in tumoral cells^6–8^. These alterations include increased glucose uptake, high glycolytic rate and pentose phosphate pathway, in conjunction with a low rate of oxidative phosphorylation through the TCA cycle^7^.

Glycolysis is the metabolic pathway that converts glucose into pyruvate. This metabolite can either be converted into lactic acid in the anaerobic respiration or into acetyl-CoA and enters the aerobic respiration through TCA cycle. Pyruvate kinase is a key enzyme in glycolysis that converts phosphoenolpyruvate and ADP into pyruvate and ATP. In the CNS, pyruvate kinase has two isoforms, PKM1 and PKM2, originated from the same gene. These isoforms are generated by a mutually exclusive alternative splicing of two exons. Under normal conditions, PKM1 is the most abundant isoform expressed in neural cells, while the expression of PKM2 is low and can be found mosty in proliferating cells, specifically in embryonic cells and neural progenitors of the subventricular zone, hippocampus and cerebellum^9^.

As a tetramer, PKM2 has similar pyruvate kinase activity as PKM1. As a dimer, PKM2 loses its pyruvate kinase activity and instead acts as transcription factor that regulates the expression of target genes involved in glycolysis [e.g. Glucose transporter 1 (Glut1), Lactate dehydrogenase A (LDHA]^10^ and inflammation^11^. Proinflammatory stimuli, such as LPS, IFNγ or TNFα, induce the transcriptional expression of PKM2, promote the dimer form, inhibiting the tetramerization and induce the phosphorylation required for nuclear translocation^7^. Through the regulation of LDHA expression and activity, PKM2 regulates NLR family pyrin domain containing 3 (NRLP3) and absent in melanoma 2 (AIM2) inflammasome activity and consequently the secretion of IL-1β and IL-18^11^. In macrophages, tetramerization of PKM2 with two small molecules, DASA-58 or TEPP-46, attenuated LPS-induced M1 proinflammatory macrophages phenotype while promoting M2 phenotype^8^.

Bile acids are cholesterol derivatives synthesized in the liver of mammals and other vertebrates and involved in the digestion of dietary fats and oils. Some bile acids, such as TUDCA, have neuroprotective^12,13^ and anti-inflammatory effects^14–16^ on several CNS pathologies. Depending on their affinity, bile acids exert their effects through the activation of the transmembrane Takeda G protein-coupled receptor 5 (TGR5) or the nuclear farnesoid X receptor (FXR). The bile acid TUDCA and a specific TGR5 agonist INT-777 have anti-inflammatory effects in microglia cells, macrophages and in a mouse model of acute neuroinflammation^17,18^. These anti-inflammatory effects depend on activation of protein kinase A (PKA) by TGR5 and are mediated by inhibition of the NFκB pathway^17,19^. Bile acids also inhibit NLRP3 inflammasome activation through TGR5-protein kinase A (PKA) axis activation^20^. This effect has been proposed as part of the mechanism responsible for bile acids inhibition of sepsis.

Here, we show that bile acids also inhibit LPS-induced PKM2 expression and its target genes in microglia, a macrophage cell line and in a rat model of SCI. These results confirm the importance of PKM2 in the inflammatory response and indicate an additional anti-inflammatory mechanism of bile acids as regulators of glycolysis.

## Methods

### Reagents

TUDCA sodium salt was purchased from Calbiochem (La Jolla, CA, USA), BAY11-7042, *Escherichia coli* lipopolysaccharides isotypes 026:B6, TLCA, RG-239 hydrate, Dulbecco’s modified Eagle's medium (DMEM), penicillin/streptomycin mix (P/S) and poly-L-lysine were purchased from Sigma-Aldrich (St Louis, MO, USA). Foetal bovine serum (FBS), GlutaMax™ and non-essential aminoacids were purchased from Gibco BRL (Gaithersburg, MD, USA). TRIzol reagent, DNase I kit, RevertAid™ H Minus First Strand cDNA Synthesis Kit and primers for qPCR were purchased from ThermoFisher Scientific (Waltham, MA, USA).

### Cell culture

Primary cultures of microglial cells, obtained from p0 to p2 Wistar rat forebrains^18^ were grown in DMEM medium supplemented with 10% heat-inactivated foetal bovine serum (FBS), GlutaMax™, non-essential amino acids (NEAA) and P/S (DMEM 10:1) in 75-cm^2^ flasks, coated with poly-L-lysine (10 μg/ml, PLL)^21^. After reaching confluency, the cells were detached in an orbital shaker at 230 rpm for 3 h (h) at 37ºC. Detached cells were centrifuged at 168×g for 10 min, plated in a non-treated culture dish and left in the incubator for 3 h. Floating cells were removed with warm PBS and attached cells (microglia) were detached with PBS with 4 mM EDTA. After centrifugation, microglia cells were resuspended in warm DMEM 10:1 and plated at a density of 200,000 cells/cm^2^. For experiments, microglial cells were resuspended in DMEM medium without phenol red supplemented with 0.5% FBS and P/S.

The mouse Raw264.7 macrophage cell line was grown in DMEM medium supplemented with 10% FBS, GlutaMax™, NEAA and P/S in cell culture dishes. For experiments, Raw264.7 cells were plated at a density of 60,000 cells/well in 96-well plates or 600,000 cells/well in 12-well plates and were cultured in DMEM without phenol red supplemented with 0.2% FBS, GlutaMax™, NEAA and P/S.

### Nitrite production

Inducible nitric oxide synthase activity was assessed in cell conditioned media^18^. Cells grown in 96-well plates or in 6-well plates were pretreated with different concentrations of bile acids for 2 h and were then treated with LPS isotype 026:B6 (10 ng/ml for microglia and 50 ng/ml for Raw264.7 cells) for 24 h in DMEM without phenol red with the corresponding concentration of FBS^18^. Supernatants (100 µL/well) were mixed (v/v, 1:1), with modified Griess reagent (10 mg/ml sulfanilamide, 1 mg/ml N-(1-naftil) ethylenediamine, 25 μg/ml of phosphoric acid dissolved in water), shaken, and absorbance was measured at 548 nm in an Infinite 200 Pro plate reader (Tecan, Männedorf, Switzerland). The concentration of unknown samples was determined through the interpolation of the absorbance in a linear standard curve of absorbance versus nitrite concentrations.

### Experimental animals

All methods have been reported in accordance with the ARRIVE guidelines (https://arriveguidelines.org). Experimental protocol, surgical procedures and post-operational care were reviewed by the ethics committee for Animal Care of the *Hospital Nacional de Parapléjicos* reviewed (163CEEA/2017) and approved by the *Consejería de Agricultura y Ganadería de Castilla-la Mancha* (ref. 210498), following EU directive 2010/63/EU.

We used 27 male wistar rats (*Rattus norvegicus*), six to eight weeks of age, which had been bred in the animal facility of the hospital. Until the day of surgery animals were kept in pairs and subsequently in individual cages. Standard housing conditions consisted in 12 h light/dark cycle, humidity 40–60%, temperature 22 °C with ad libitum access to food and water.

### Surgical procedures and postoperative treatment

For experimental spinal cord contusion injury, anesthesia was induced with 5% isoflurane/95% oxygen, flow rate 0.4 L/hour in a respirator, and maintained with 2.5% isoflurane. Fifteen minutes before surgery, rats were received one subcutaneous (s.c.) injection of buprenorfine 0.05 mg/kg to reduce pain. Corneal dehydration was prevented with ophthalmic ointment (Lubrithal). Following laminectomy at thoracic level T8–10, a spinal cord contusion of 2 N (200 Kdyn, zero dwell time) was inflicted with the Infinite Horizon spinal cord impactor. The control group was operated using the laminectomy procedure without SCI. Immediately after surgery, all animals received 2 × 2.5 ml isotonic saline s.c. and antibiotic treatment marbofloxacine 5 mg/kg (Marbocyl 10 mg/mL, s.c.). Control of surgery and postoperative care, including analgesia, antibiotic treatment and manual voiding of the bladders were done as previously described^22^. At 4 days post operation (dpo), euthanasia was induced by intraperitoneal (i.p.) injection of sodium pentobarbital (Dolethal).

### Experimental groups

Animals were assigned to three experimental groups. One group (sham control, *n* = 9) had T8-10 laminectomy but no contusion injury. Group 2 (SCI-NaCl control, *n* = 9) received two NaCl i.p. injections, the first immediately after SCI (t0) and the second 24 h later (1 dpo). Group 3 (SCI-TUDCA, *n* = 9) was treated with two i.p. injections of TUDCA 300 mg/kg body weight at t0 and 1 dpo. In each group, 5 animals were randomly assigned for biochemical analysis of spinal cord extracts, and 4 rats were perfused to perform IHC experiments. Complying with the principles of 3Rs in animal research, these rats formed part of a larger study about the therapeutic effect of bile acids after SCI (unpublished data).

### Quantification of gene expression

Animals for biochemical evaluation were sacrificed at 4 dpo with an overdose of sodium pentobarbital. After opening the thoracic cavity, rats were perfused with phosphate buffered saline (PBS; 200 ml/rat, 25 ml/min). Spinal cord samples, which consisted of a 2 cm segment with the lesion site in the center, were prepared for biochemical analysis^22^.

Total RNA was extracted from cell cultures or mechanically homogenized tissue with Trizol reagent (Invitrogen, 15596018) according to manufacturer’s instructions. To remove genomic DNA, purified RNA was digested with DNase I (ThermoScientific, EN0521). An aliquot corresponding to 2 μg of purified RNA was used for first-strand cDNA synthesis using Superscript III reverse transcriptase and oligo (dT) primers in a final volume of 40 μl (Invitrogen Life Technologies, K1632)^23^. Real-time quantification of genes was performed using a SYBR Green RT-PCR assay. Each 15 μl SYBR green reaction mixture consisted of 1 μl cDNA, 7.5 μl with qPCRBIO SyGreen Mix Hi-Rox (PCR Biosystems, Wayne, USA) (2×), 0.75 μl forward and reverse primer (5 µM) and 4.75 μl distilled water. PCR was performed with 2 min at 95ºC, followed by 40 cycles of 15 s at 95ºC, 30 s at 60 °C and a separate dissociation step^23^. Specificity of the PCR product was confirmed by ascertaining a single melting peak in the temperature dissociation plots. All samples were run in duplicates and the level of expression of each gene was compared with the expression of acidic ribosomal phosphoprotein P0 (36B4) for microglia cultures and tissue samples, and RPS29 for Raw264.7 cells respectively^23^. Amplification, detection of specific gene products and quantitative analysis were performed using an ABI 7900HT sequence detection system (Applied Biosystems, USA). PCR efficiency was verified by dilution series (1, 1/3, 1/9, 1/27, 1/81 and 1/243) and relative mRNA levels were calculated using the comparative ΔΔCt method with normalization to 36B4 or RPS29^23^. Gene identifiers, primer sequences and product sizes are listed in Table 1.

**Table 1:** Gene identifiers and primer sequences used in quantitative RT-PCR.

Gene	Gene ID (NCBI reference sequence)	Primer sequences	Product size [bp]
Rat 36B4	NM_022402.2	Sense: TTCCCACTGGCTGAAAAGGT Antisense: CGCAGCCGCAAATGC	59
Mouse/Rat Glut1	NM_011400.3 NM_138827.2	Sense: GCTCAGTGTCATCTTCATCCCAG Antisense: AGGTCTCGGGTCACATCGG	155
Mouse/Rat HMGB1	NM_153198.3 NM_013221.2	Sense: CAAATGGGCACTCACAAGGG Antisense: ACTCACCGAATGACACACTCT	78
Rat IFNβ	NM_019127.1	Sense: CAAAGCACTAGCATTCGGACAT Antisense: TGAGGTTGAGCCTTCCATTCA	63
Rat IL-6	M26745.1	Sense: TAGTCCTTCCTACCCCAATTTCC Antisense: TTGGTCCTTAGCCACTCCTTC	75
Rat IL-1β	NM_031512.2	Sense: GACTTCACCATGGAACCCGT Antisense: GGAGACTGCCCATTCTCGAC	103
Mouse iNOS	M92649.1	Sense: ACATTGATCTCCGTGACAGCC Antisense: CCCTTCAATGGTTGGTACATG	157
Mouse LDHA	BC094019.1	Sense: GTTGTTGGGGTTGGTGCTGT Antisense: TCATCTCGCCCTTGAGTTTGT	114
Rat LDHA	NM_017025.2	Sense: GCACTAAGCGGTCCCAAAAG Antisense: ACAGCACCAACCCCAACAAC	125
Mouse/Rat PGK1	NM_008828.3 XM_032890039.1	Sense: GCCTGTTGACTTTGTCACTGC Antisense: AGCAGGTATACCAGAGGCCA	81
Mouse PKM1	NM_001253883	Sense: AGCCTCCAGTCACTCCACAGA Antisense: TCAGCACGGCATCCTTACAC	238
Rat PKM1	M24359.1	Sense: AGCCTCCAGTCAATCCACAGA Antisense: ACGGCATCCTTACACAGCACA	234
Mouse/Rat PKM2	NM_011099.4 M24359.1	Sense: ATTACCAGCGACCCCACAGAA Antisense: ACGGCATCCTTACACAGCACA	224
Mouse RPS29	NM_ 009,093.2	Sense: GCCGCGTCTGCTCCAA Antisense: ACATGTTCAGCCCGTATTTGC	54

### Tissue preparation and histological staining

At four days after SCI, rats were sacrificed with an overdose of sodium pentobarbital followed by transcardial perfusion with PBS and 4% paraformaldehyde/PBS (PFA)^22^. Spinal cords were prepared, post-fixed for 1 h then stored at 4 °C in PFA for 3 days. For histological processing, 2 cm long spinal cord segments that included the lesion site in the center were dissected, dehydrated, embedded in paraffin and cut in 3 μm parasagittal sections using a Leica RM2265 microtome^22^. Sections were mounted on PLL-coated glass slides (Superfrost Plus) and stored at room temperature (RT).

### Immunohistochemistry

For immunohistochemical (IHC) staining, sections were rehydrated and incubated in for 30 min at 90 ºC (water bath) in 10 mM Na citrate/0.05% Tween 20, pH 6.0, for antigen retrieval^22^. Standard procedure included blocking 30 min at RT with 5% normal goat serum (NGS)/0.1% Tween 20 in Tris-buffered saline (TBS-T), incubation with primary antibodies (2% NGS) for 12 h at 4 ºC in a humidified chamber and 1 h incubation with fluorescence-labeled secondary antibodies at RT^22^. Nuclei were stained with 10 μg/mL Hoechst-33342 for 15 min at RT. Sections were cover slipped with ImmuMount (Thermoscientific). The following primary antibodies were used in double staining experiments: Guinea pig anti-Iba1, polyclonal (Synaptic systems 234004; 1/500) and rabbit anti-PKM2 (Cell Signaling; 4053, 1/500). Secondary antibodies were labeled with fluorescent dyes: Goat anti-guinea pig IgG, Alexa-488 (Invitrogen A11073; 1/500), and goat anti-rabbit IgG, Alexa594 (Invitrogen A11005; 1/1000).

### Microscopy and image analysis

Immunohistochemical staining was evaluated using a Leica epifluorescence microscope^22^. Exposure conditions were kept constant for quantitative evaluation with (20 × objective) or (40 × objective) Iba1 and PKM2 staining. Photographs were analyzed using Fuji Image-J, applying the same brightness/contrast adjustments and threshold values for each marker. Signal intensities were normalized to values found in spinal cord sections from sham-operated rats^22^. The intensity of immunoreactivity was measured as *integrated density* in regions of interest (ROI 0.3 mm^2^) in the ventral white matter at 0.8 cm distance anterior and posterior of the lesion center. Iba1 and PKM2 positive cells were counted in the same ROIs and within the lesion center (ROI 0.075 mm^2^). For the evaluation of cellular expression of PKM2, we calculated the percentages of Iba1/PKM2 double stained cells within the Iba1 positive cell population (microglia/macrophages), and of Iba1 positive cells that showed nuclear PKM2 staining.

### Statistical analysis

GraphPad Prism software version 5.0 for Windows was used for statistical analysis and to create the graphs. Depending on the experiment, the variances of the treatments were compared with one-way ANOVA and the statistical significance between groups was determined by Dunnett’s or Bonferroni post-hoc tests. Data in graphs are presented as the mean ± SEM. In the SCI experiment, data from individual rats were considered as independent data.

## Results

### Bile acids reduce the expression of PKM1/PKM2 with LPS treatment

**Figure 1:**
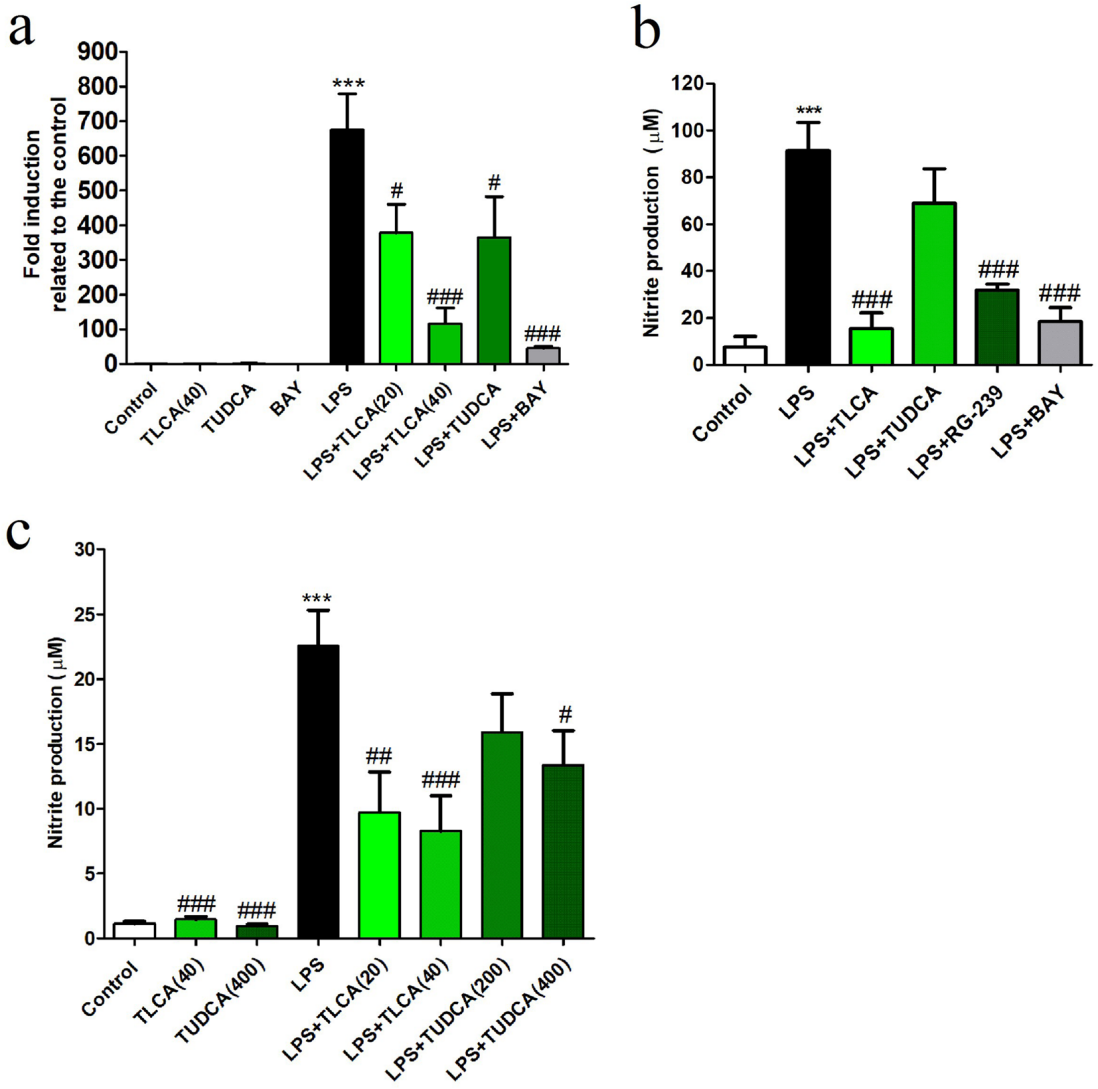
Bile acids inhibit LPS-induced nitrite production in cell cultures. **(a)** Transcript expression of iNOS was determined in Raw264.7 cells, pretreated with TLCA (20 or 40 µM), TUDCA (200 µM) or BAY11-7042 (5 µM) for 2 h; LPS (50 ng/ml) was added and incubated for 24 h. Concentrations of iNOS transcripts were normalized to expression of RPS29 and the control condition without LPS (mean ± SEM, *n* ≥ 4 experiments). ANOVA revealed significant treatment effects on iNOS expression [F (7, 31) = 11.9, *p* < 0.001). **(b)** Nitrite production (in µM) by Raw264.7 cells was strongly induced by LPS and significantly inhibited by TLCA and the specific TGR5 agonist RG-239 (2 µM) [F (5, 20) = 15.0), *p* < 0.001]. **(c)** In microglia primary cultures, nitrite release (µM) was also induced by LPS (10 ng/ml) and inhibited by bile acids [F (7, 73) = 15.9, *p* < 0.001]. Statistical significance of post hoc Dunnett's tests is indicated significance between treatments and the controls ***p < 0.001 (control vs. LPS) and # *p* < 0.05, ##*p* < 0.01 and ###*p* < 0.001 (LPS vs. LPS + treatment).

We used LPS proinflammatory pathway to validate the effect of several bile acids on iNOS expression and nitrite production in the Raw264.7 macrophage cell line (Fig. 1a, b) and rat microglial cells (Fig. 1c). TUDCA inhibited LPS-induced iNOS expression (Fig. 1a) and nitrite production (Fig. 1b) in Raw264.7 and microglia cells. TLCA and the TGR5 agonist RG-239 had the same effect as TUDCA in both type of cells at lower concentration.

**Figure 2:**
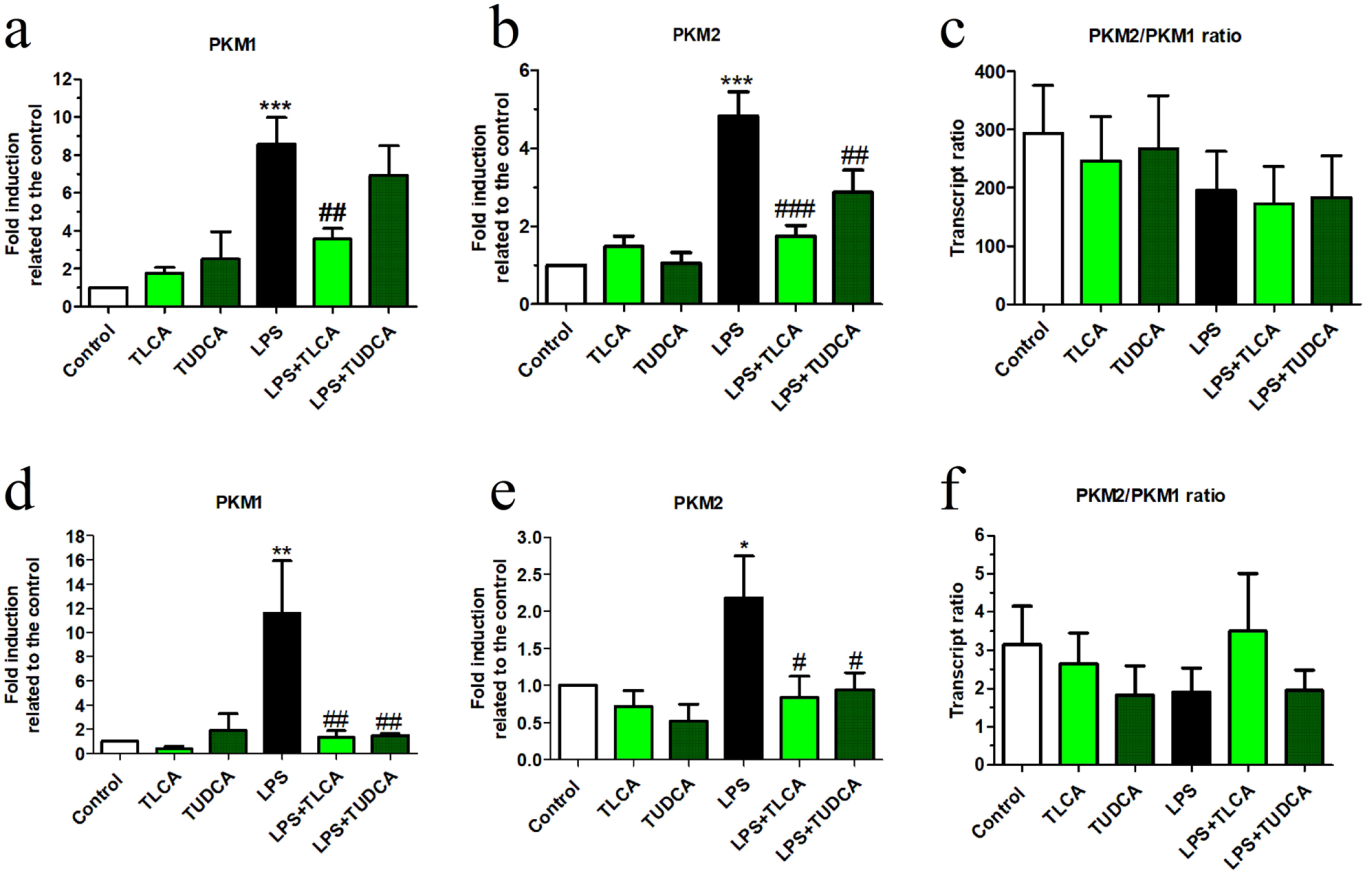
Treatment with bile acids inhibits LPS-induced PKM1 and PKM2 transcription in cell cultures. The mRNA expression for **(a, c)** PKM2 and **(b, d)** PKM1, and **(e)** PKM2/PKM1 transcript ratio was determined by qRT-PCR in microglia and in Raw264.7 cultures. Cells were preincubated with TLCA (40 μM), TUDCA (200 μM) for 2 h followed by LPS (10 ng/ml for microglia and 50 ng/ml for Raw264.7 respectively) was added for further 24 h. The expression of 36B4  for microglia or RPS29 for Raw264.7 cells respectively was used as a housekeeping gene to normalize the data. ANOVA revealed significant treatment effects on the transcription of PKM1 in **(a)** microglial cells [F(5, 28) = 9,3, *p* < 0.001] and **(d)** in Raw264.7 cells, [F(7, 21) = 4.7 *p* < 0.01] and PKM2 **(b)** in microglial cells [F(5, 29) = 13.7, *p* < 0.001] and **(e)** in Raw264.7 cells [F(5, 23) = 3.7, *p* < 0.05] respectively. Changes of PKM2/PKM1 ratio in **(c)** microglial cells and **(f)** Raw264.7 cells were not statistically significant. Results of Dunnett’s post hoc tests are indicated as in Fig. 1 (* vs. control, # vs. LPS, *n* ≥ 3 independent experiments).

Inflammatory pathways induce an increase of glucose metabolism^7^. The enzyme isoforms PKM1 and PKM2 catalyze the conversion of phosphoenolpyruvate into pyruvate, which is a rate-limiting reaction of glycolysis. We found that both isoforms were transcriptionally induced by LPS in rat microglia cells and Raw264.7 macrophages (Fig. 2). LPS transcriptional induction of both isoforms was inhibited by bile acids in microglia cells (Fig. 2a, b) and in Raw264.7 macrophages (Fig. 2d, e). The transcriptional ratio between PKM2 and PKM1 in both cell types (Fig. 2c, f), remained stable with the treatments, suggesting that LPS and bile acids effects were at the transcriptional level and did not affect differential splicing.

### Bile acids reduce the upregulation of PKM2 target genes

**Figure 3:**
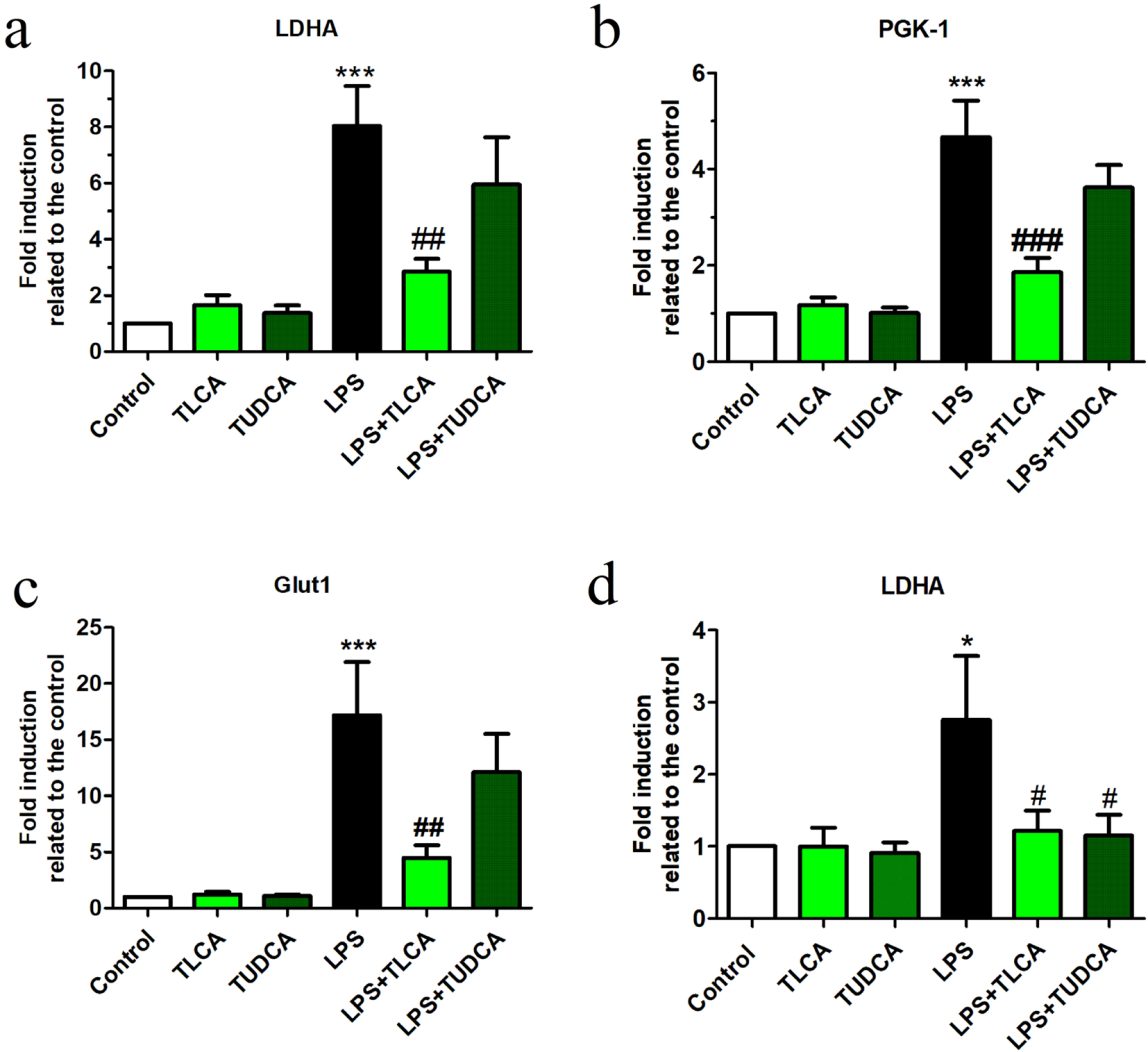
Bile acids inhibit LPS-induced transcription of PKM2 target genes in cell cultures. The mRNA expression for **(a, b)** LDHA, **(c)** PGK-1 and **(d)** Glut1 was determined by qPCR in microglia and in Raw264.7 cultures. Cells were preincubated with TLCA (40 μM), TUDCA (200 μM) for 2 h followed by LPS (10 ng/ml for microglial cell and 50 ng/ml for Raw264.7 cells) for further 24 h. Normalization of qRT-PCR data was done as in Fig. 2 (ΔΔCt, 36B4 for microglia, RPS29 for Raw264.7 cells). ANOVA revealed significant treatment effects on the transcription of LDHA **(a)** in microglial cells [F (5, 23) = 8.9, *p* < 0.001} and **(d)** in Raw264.7 cells [F(5, 25) = 2.9, *p* < 0.05], PGK-1 **(b)** [F(5, 23) = 14.9, *p* < 0.001] and Glut1 **(c)** [F(5, 23) = 7.5, *p* < 0.001)]. Results of Dunnett’s post hoc tests are indicated as in Fig. 1 (* vs. control, # vs. LPS, *n* ≥ 3 independent experiments).

As a transcription factor, PKM2 regulates the expression of several glycolytic enzymes such as LDHA, Glut1 and phosphoglycerate kinase-1 (PGK-1)^24^. We checked whether bile acids had an effect on PKM2 function as a transcription factor. LPS increased the expression of all the mentioned PKM2 target genes in microglial cells (Fig. 3a-c) and LDHA in Raw264.7 cells (Fig. 3d). TLCA significantly inhibited the transcription of Glut1, PGK-1 and LDHA. TUDCA also reduced the expression of these target genes though this reached statistical significance only in Raw264.7 cells with respect to LDHA (Fig. 3d).

**Figure 4:**
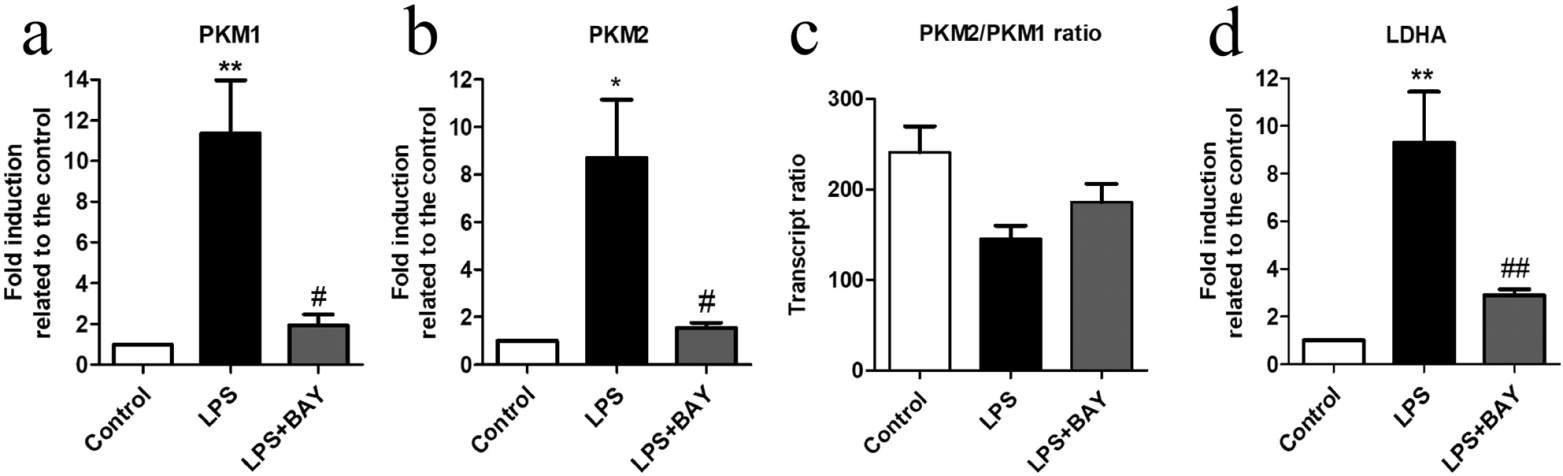
BAY11-7042, an inhibitor of NFκB, reduced PKM1, PKM2 and LDHA transcripts in microglial cells treated with LPS. The mRNA expression for **(a)** PKM1, **(b)** PKM2 and **(d)** LDHA was determined by qPCR in microglia cultures. Cells were preincubated with BAY11-7042 (2 μM) for 1 h and then LPS (10 ng/ml) was added for further 24 h. The expression of 36B4 was used as a housekeeping gene to normalize the data. Results represent the mean ± SEM of fold induction related to the control of the target gene transcript expression and 36B4 transcript expression of at least *n* ≥ 3 independent experiments. ANOVA revealed significant treatment effects on the transcription of **(a)** PKM1 [F (2, 10) = 11.3, *p* < 0.01]; **(b)** PKM2 [F(2, 10) = 7.9, *p* < 0.01] and **(c)** LDHA [F(2, 6) = 27.8, *p* < 0.001]. **(d)** Changes of PKM2/PKM1 ratio were not statistically significant. Bonferroni post hoc test revealed significance between same experimental groups and the controls * *P* < 0.05, ** *P* < 0.01 and # *p* < 0.05, ## *p* < 0.01 and compared to LPS treatment.

Since activation of TGR5 inhibits the NFκB pathway^17,19^, we tested whether this mechanism was also involved in the transcriptional regulation of PKM. BAY11-7042, an inhibitor of NFκB, reduced the LPS-dependent PKM1 (Fig. 4a), PKM2 (Fig. 4b) and LDHA transcripts (Fig. 4d) in microglial cells. However, inhibition of NFκB did not have any effect on PKM1/PKM2 ratio (Fig. 4c). These results suggest that bile acids might be attenuating the expression of PKM2 and target genes through the inhibition of NFκB pathway.

### TUDCA attenuates the expression of PKM2 and its target genes after SCI

**Figure 5:**
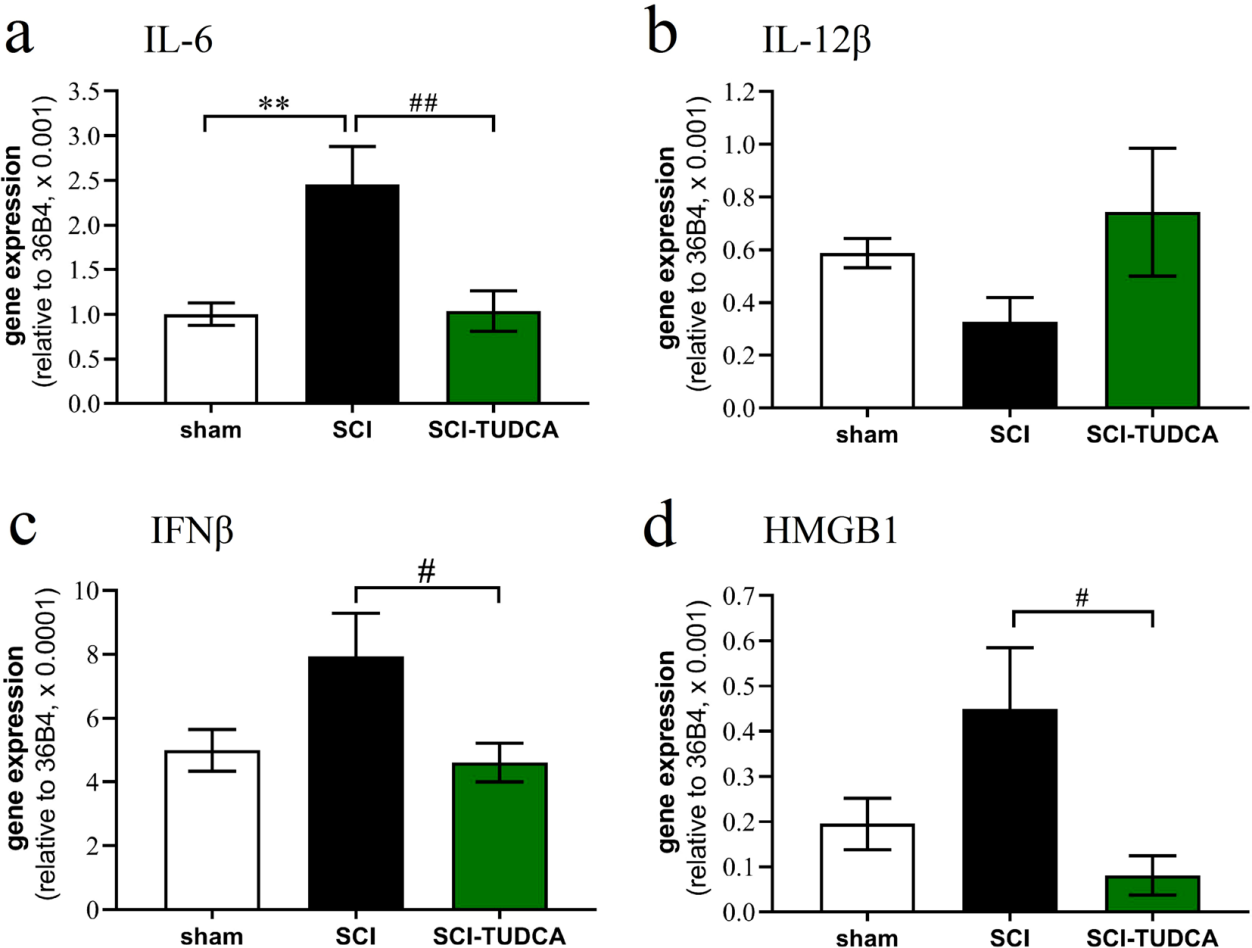
Treatment of SCI with TUDCA reduces expression of inflammatory genes. At 4 dpo, RNA extracts from spinal cord were analyzed with quantitative RT-PCR. ANOVA revealed significant treatment effects on the transcription of **(a)** IL-6 [F (2, 12) = 8.3, *p* < 0.01, **(b)** IL-12β [F (2, 12) = 1.6, n.s.]; **(c)** IFNβ [F (2, 12) = 3.9, *p* < 0.05], and **(d)** HMGB1 [F (2, 12) = 4.5, *p* < 0.05]; post hoc Dunnett's tests: * comparison SCI vs. control; # SCI vs SCI-TUDCA; *n* = 5 rats). Treatments conditions are indicated as sham: laminectomy only; SCI: SCI with two i.p. injections of saline; SCI-TUDCA: SCI with two injections of 300 mg/kg TUDCA at the time of surgery and 24 h later.

TUDCA has been shown to reduce acute inflammation *in vivo*^18^. To investigate whether this anti-inflammatory activity extends to the regulation of PKM2 and glycolysis related genes in vivo, we studied the mechanism in a rat contusion model of SCI with survival time of 4 days. Bile acid treatment consisted of two i.p. injections of 300 mg/kg TUDCA. This experiment confirmed the anti-inflammatory effect of TUDCA on expression of cytokine IL-6 (Fig. 5a), though not IL-12β (Fig. 5b). In addition, we found that the SCI-induced upregulation of IFNβ and HMGB1 was completely inhibited by TUDCA (Fig. 5c, d).

**Figure 6:**
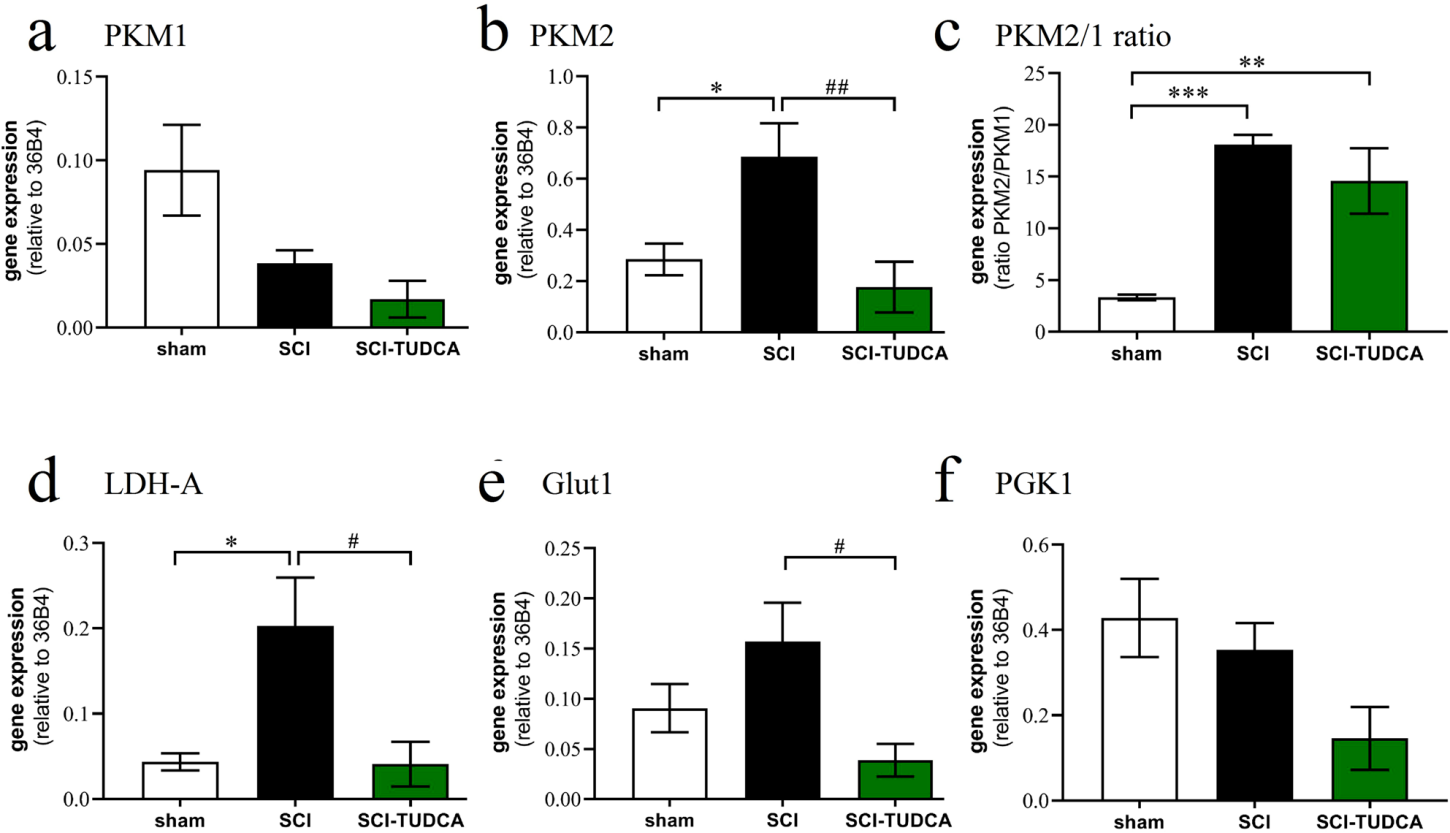
Treatment of SCI with TUDCA reduces expression of genes involved in glucose metabolism. At 4 dpo, RNA extracts from spinal cord were analyzed with quantitative RT-PCR. ANOVA revealed significant treatment effects on the transcription of **(a)** PKM1 [F (2, 12) = 5.2, *p* < 0.05, post-hoc Dunnett's tests n.s.], **(b)** PKM2 [F (2, 12) = 7.0, *p* < 0.01], **(c)** mRNA ratio PKM2/PKM1 [F (2, 12) = 16.2, *p* < 0.001]; **(d)** LDH-A [F (2, 12) = 6.3, *p* < 0.05]; **(e)** Glut1 [F (2, 12) = 5.1, *p* < 0.05]; and **(f)** PGK1 [F (2, 12) = 5.2, *p* < 0.05, post-hoc tests n.s.]; Results of post hoc Dunnett’s tests and treatment conditions are indicated as in Fig. 5 (* vs. control, # vs. LPS, *n* ≥ 5 rats).

Similarly, SCI caused a significant upregulation of PKM2 and target genes LDHA and Glut1. The expression of PKM1 and PGK-1 were not significantly affected by SCI (Fig. 6). In rats treated with TUDCA the upregulation of PKM2, LDHA and Glut1 was completely inhibited. Due to the increase of PKM2 after lesion but not of PKM1, the PKM2/PKM1 transcriptional ratio also rose significantly. Treatment affected the concentration of both mRNA species to a similar degree, suggesting that the bile acid inhibited transcription of PKM in the injured spinal cord, but had no influence on the splicing of this gene.

### Altered expression of PKM2 in microglia and macrophages after SCI

Previously, PKM2 was detected in astrocytes in the spinal cord^25^. To relate the PKM2 data of microglia and macrophages in vitro with effects on gene expression after SCI we performed double IHC experiments using a PKM2 antibody in combination with Iba1 for microglia/macrophage. We found PKM2 immunoreactivity in neurons, astrocytes and in microglia cells. Among the microglia population, the percentage of Iba1 positive cells (49% + /− 23%, mean + /− SD) was higher than in the cell population at large (32% ± 14%, all cells in grey and white matter combined). In sham-operated rats a quarter of all PKM2 positive cells were microglia.

**Figure 7:**
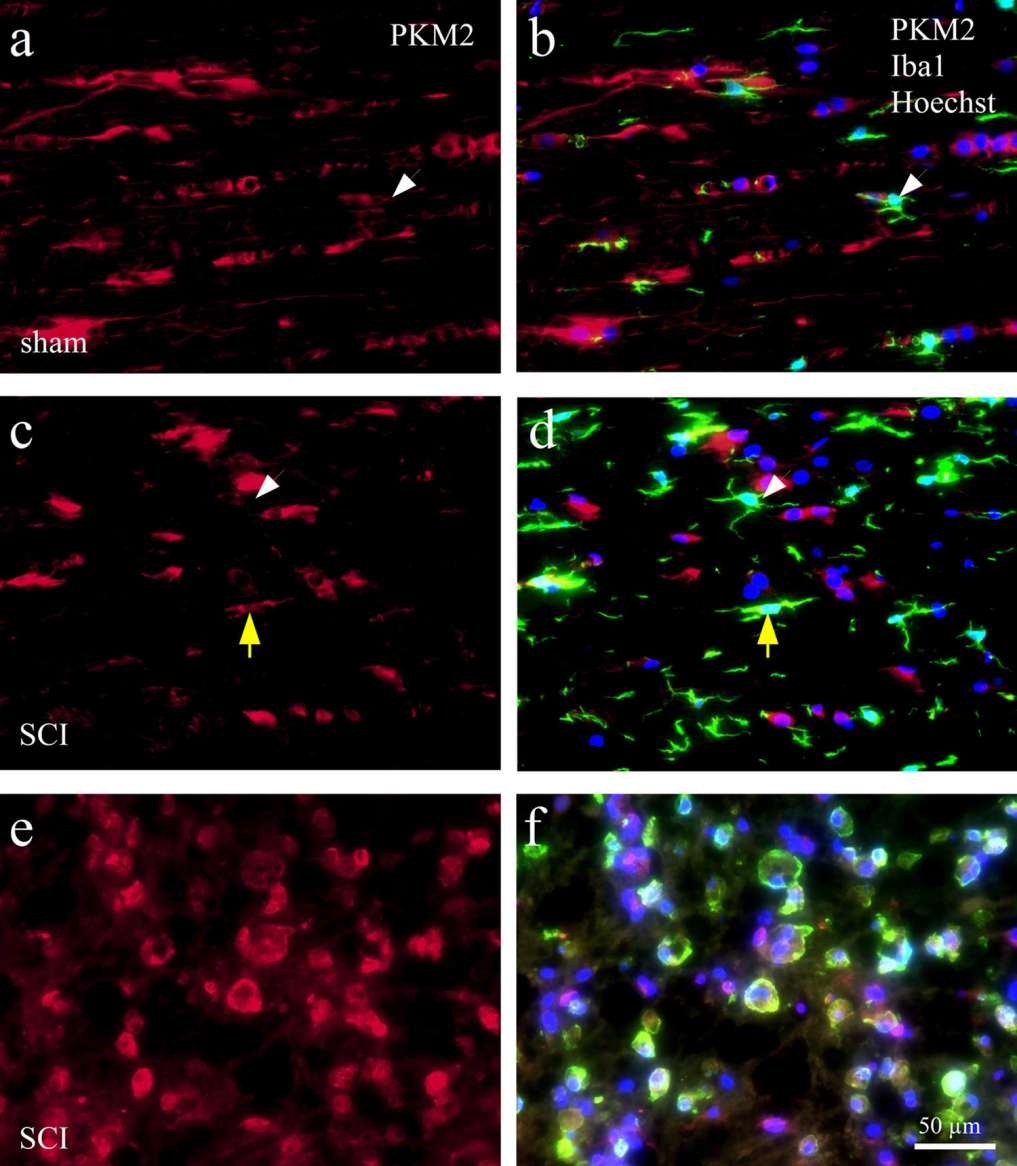
Immunoreactivity of PKM2 in glia cells in the rat spinal cord. IHC staining with an antibody against PKM2 (a, c, e, red fluorescence) was combined with Iba1 (green) and Hoechst nuclear stain (blue, triple labeling shown in b, d, f). **(a, b)** PKM2 immunoreactivity in dorsal white matter of a rat without SCI; **(c, d)** PKM2 staining in dorsal white matter at 4dpo and 8 mm distance from the lesion center; white arrow heads point at microglia without PKM2 staining, and yellow arrows indicate a microglia cell with nuclear PKM2 staining. **(e, f)** PKM2 immunoreactivity at 4dpo in the lesion center; many Iba1 positive cells display PKM2 staining in their nuclei (violet staining in **f**). Scale bar in f applies to all panels.

At 4 days after spinal cord injury, the number and labeling intensity of Iba1 cells had increased in the white and grey matter surrounding the lesion site. Many of these cells expressed PKM2 (Fig. 7a, c, e). Within the lesion center most of Iba1 cells were also positive for PKM2 (Fig. 7b, d, f), and the enzyme was often located in their cell nuclei. Of the PKM2 positive cells in the lesion center 68% co-localized for Iba1.

**Figure 8:**
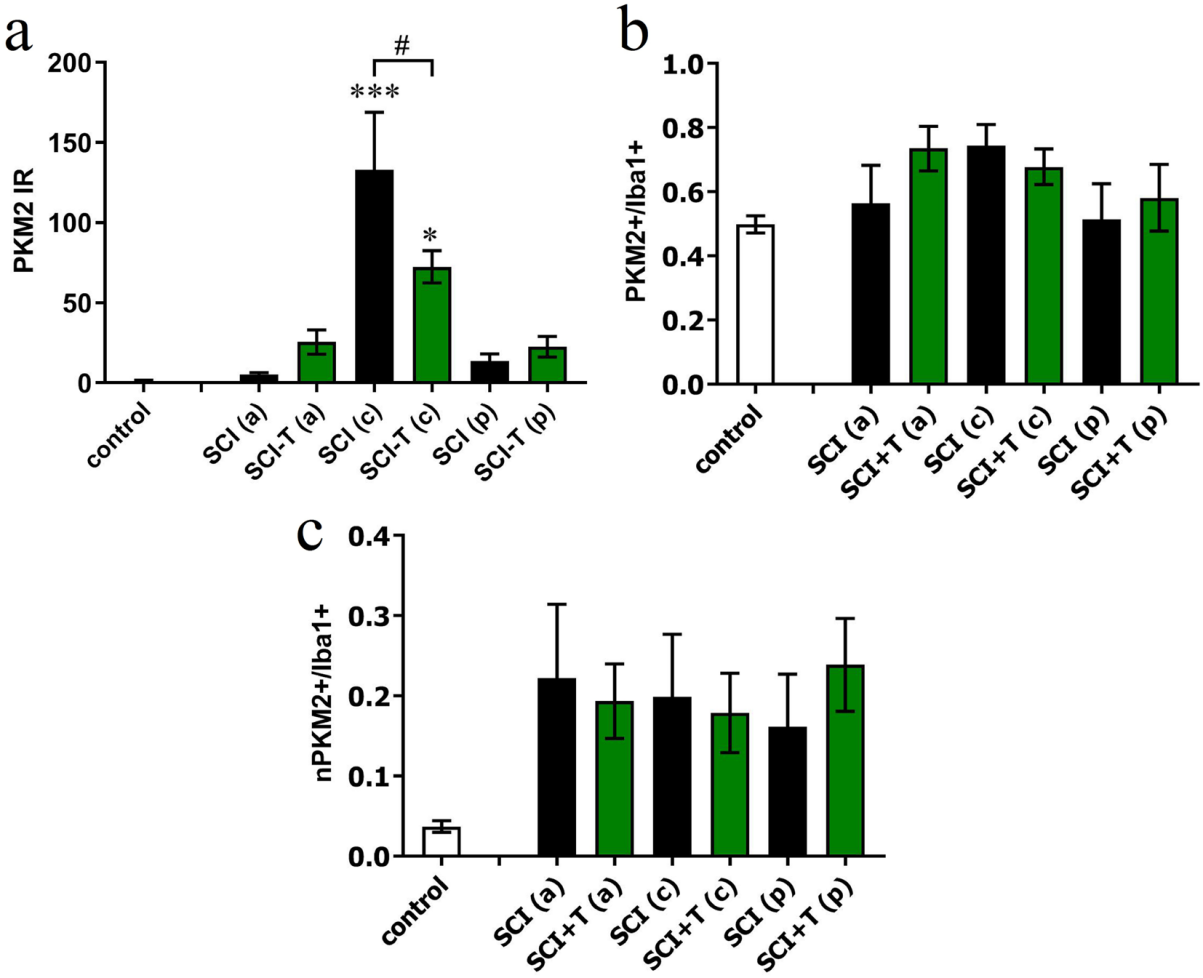
Changes of PKM2 IR after SCI and treatment with TUDCA. **(a)** Quantification of PKM2 immunoreactivity (integrated density) at 4 dpo in white matter of rats with laminectomy (control), at 8 mm anterior (SCI a), posterior (SCI *p*) or in the lesion center (SCI c) shows a highly significant increase in the lesion center, which was lower in TUDCA treated animals. ANOVA revealed significant treatment effects [F (6, 33) = 11.3, *p* < 0.001]. **(b)** In the lesion center, the proportion of Iba1 positive microglia/macrophages that expressed PKM2 increased, less so after TUDCA treatment [F (2, 11) = 6.4, *p* < 0.05]. **(c)** The proportion of Iba1 positive cells with PKM2 labeling within their nuclei was higher at all locations after SCI irrespective of TUDCA treatment [although not significantly: ANOVA, F (6, 25) = 1.1, n.s.]; results of post hoc Dunnett's test in panels a, b: * for comparison SCI vs. control, # for SCI vs SCI-TUDCA.

Quantitatively the PKM2 immunoreactivity was very low in the sham group and increased dramatically after SCI, where it was much higher in the center of the lesion than in anterior or posterior white matter. In accordance with the PCR data, TUDCA treatment significantly reduced the IR levels of PKM2 (Fig. 8a). When comparing sham operated rats with those that received a SCI, we did not find that the proportion of PKM2 positive cells among the Iba1 population changed significantly (Fig. 8b). However, in the lesion center, a larger proportion of the Iba1 microglia/macrophages were also PKM2 positive than among the microglia cells in white matter of control animals (*p* < 0.05). The percentage of PKM2/Iba1 positive cells that displayed this protein in their nuclei increased after SCI, indicating that PKM2 is recruited as a transcription factor under inflammatory conditions (Fig. 8c). Treatment with TUDCA did not affect the cellular distribution of PKM2 within Iba1 positive cells.

## Discussion

In this study, we investigated whether bile acids inhibit the PKM1/PKM2 pathway under inflammatory conditions. The results suggest three conclusions: (1) Following treatment with LPS, the expression of PKM1, PKM2, LDHA, Glut1 and PGK-1 increased in microglia cultures. Treatment with bile acids, most significantly TLCA, inhibited this effect, but did not alter its splicing. (2) In the acute phase after SCI, the expression of PKM2, LDHA and Glut1 was increased close to the lesion site. Intrathecal injections of 300 mg/kg TUDCA blocked this effect without affecting the PKM2/PKM1 ratio. PKM2 protein was located in Iba1 immunoreactive macrophages in the lesion center, and TUDCA treatment reduced the lesion-induced increased of PKM2 immunoreactivity. (3) The effects of bile acids in vitro could be mimicked inhibiting NFĸB signaling. Since this pathway is known to be affected by TGR5 activation, we suggest that TUDCA and TLCA regulate PKM1, PKM2 and target genes expression through the inhibition of NFĸB.

Proinflammatory macrophages undergo metabolic changes to satisfy their high demand for energy and biosynthetic precursors (e.g. proteins, lipids and nucleic acids)^6^.

Similar to cancer cells, proinflammatory macrophages show increased glucose uptake, higher glycolytic rate and pentose phosphate pathway, together with a lower rate of oxidative phosphorylation through TCA cycle (Warburg effect)^26^. Pyruvate kinase is a key enzyme in the regulation of the Warburg effect. In resting macrophages, PKM1 is the most abundant isoform with highest pyruvate kinase activity converting efficiently phosphoenolpyruvate to pyruvate to fuel both glycolytic pathway and TCA cycle. Proinflammatory cytokines increase the expression of PKM2 and inhibit the tetramerization, swtiching this equilibrium to increase glycolysis in macrophages. As a monomer/dimer, PKM2 is a transcription factor that upregulates the expression of the glucose transporter Glut1, increasing glucose uptake. Moreover, PKM2 upregulates the expression of PGK-1 and LDHA, increasing lactate production and reducing the availability of pyruvate for TCA cycle^24^.

In the CNS; PKM1 is the most abundant isoform under normal conditions and it is constitutively expressed in neural cells^9^. PKM2 was reported to be expressed specifically in proliferating cells such as neural precursors of the subventricular zone and hippocampus^9^. After SCI, there is an increase in PKM2 expression in the injured area, mainly in proliferating astrocytes indicating that PKM2 expression may be regulating cell cycle progression in astrocytes^25^. While there is extensive information about PKM2 expression in proinflammatory macrophages^27^, little is known about its role in microglial cells. Proinflammatory cytokines increase the transcription and expression of PKM2, but not PKM1 in macrophages^7,8^. We found that the expression of both PKM1 and PKM2 transcript is induced by LPS in microglial cultures and Raw264.7 cells. Corroborating its role in inflammation, we found the expression of PKM2 increased after SCI. In vivo, the concentration of PKM1 mRNA was not altered. This difference between LPS treated cell cultures and in vivo experiments might be affected by the cell heterogenicity of the spinal cord tissue. Interestingly, the PKM2/PKM1 ratio remained similar after bile acid treatments in vivo and in vitro*,* suggesting that gene splicing was not affected.

Our IHC investigation of the rat spinal cord revealed the presence of PKM2 in a high percentage of microglia cells. In the acute phase following SCI the PKM2 immunoreactivity increased in Iba1labeled microglia/macrophages in the lesion site. In this area, bile acid treatment reduced PKM2 labeling. Intriguingly, at 4 days after lesion more microglia and macrophages showed a nuclear localization of PKM2 than was observed in microglia in the non-injured spinal cord, indicating that PKM2 is recruited as a transcription factor under the inflammatory condition. This finding is in accordance with the increased proportion of PKM2 dimers in LPS treated microglia. However, since TUDCA treatment did not significantly affect the intracellular distribution of PKM2 in vivo, and the biochemical data are only preliminary, this mechanism remains to be investigated.

Proinflammatory cytokines induced the activation of PKM2 transcription, dimerization and the transport into the nucleus to increase the transcription of its target genes in macrophages^7^. The mechanism of these effects involves activation of NFκB and binding to a consensus sequence in the PKM promoter^28^. In this study, we found that the treatment with bile acids or an inhibitor of NFκB (BAY11-7042) reduced the expression of PKM1, PKM2 and the target genes in cell cultures. Moreover, TUDCA treatment reduced the expression of PKM2, target genes and proinflammatory cytokines in the injured spinal cord. We have previously shown that TUDCA reduced the proinflammatory response through the inhibition of NFκB activity^18^. These results suggest that the inhibition of NFκB activity by bile acids might be involved in PKM1/PKM2 pathway attenuation in proinflammatory microglial cells.

The inhibitory effect of bile acids on the inflammatory pathway has been related to the induction of cAMP levels through TGR5 receptor activation^29^ which is a G-protein coupled receptor that activates PKA via the adenylate cyclase/cAMP. The anti-inflammatory effect of cAMP and its synthetic analogs have been extensively reported in macrophages^30^. TUDCA inhibits NFκB activity through the activation of TGR5 receptor in microglial cells^18^. Pretreatment with a cAMP competitor (Rp-cAMPS) rescues the effect of TUDCA on NFκB transcriptional activity in microglial cells^19^. However, at the same time, PKA activation is required for full activation of NFκB pathway^31,32^.

There is evidence that bile acids might be regulating NFκB pathway indirectly through the inhibition of the inflammasome activity^20^. PKA, activated by TGR5, phosphorylated NLRP3 protein that inhibits the capacity of NLRP3 to interact with other inflammasome components, reducing the production of mature IL-1β^20^. PKM2 has been postulated to be a key player in inflammasome induction by proinflammatory stimuli^11^. The activation of PKM2 increased LDHA expression, raising lactate production, that finally activates the inflammasome and the production of mature IL-1β and the release of other proinflammatory mediators (HMGB1)^11^. Both mechanisms may be involved in the effect of bile acids on inflammasome activation and consequently in their effect on the activation of NFκB. Further experiments will be conducted to examine the effect of TUDCA on inflammasome formation in SCI.

In conclusion, we found that bile acids attenuate the PKM1/PKM2 pathway in LPS-treated microglia cultures and in the injured spinal cord. Our results indicate a new role of bile acids as inhibitor of the glycolytic pathway in proinflammatory microglia.
