# Author Correction: Bile acids attenuate PKM2 pathway activation in proinflammatory microglia

**DOI:** 10.1038/s41598-022-07497-6

**Published:** 2022-02-23

**Authors:** Lorenzo Romero-Ramírez, Concepción García-Rama, Siyu Wu, Jörg Mey

**Affiliations:** 1 Laboratorio de Regeneración Nerviosa e Inmunidad Innata, Hospital Nacional de Parapléjicos, SESCAM, Finca la Peraleda s/n, 45071 Toledo, Spain; 2 School of Mental Health and Neuroscience and EURON Graduate School of Neuroscience, Maastricht University, Maastricht, The Netherlands

Correction to: *Scientific Reports* https://doi.org/10.1038/s41598-022-05408-3, published online 27 January 2022

The original version of this Article contained an error in the Funding section.

“The project was funded by the Ministerio de Economía y Competitividad, Spain, SAF2017-89366R. SW received a scholarship from the Chinese Scholarship Council, Grant/Award Number 201606300031.”

now reads:

“The project was funded by SAF2017-89366R (MINECO/AEI/FEDER/UE). SW received a scholarship from the Chinese Scholarship Council, Grant/Award Number 201606300031.”

The original Article has been corrected.

